# Perspectives of caregivers towards physiotherapy treatment for children with burns in Harare, Zimbabwe: A cross-sectional study

**DOI:** 10.1186/s41038-016-0057-5

**Published:** 2016-12-02

**Authors:** Matthew Chiwaridzo, Vimbayinashe Juliet Zinyando, Jermaine Matewu Dambi, Farayi Kaseke, Nyaradzai Munambah, Tapfuma Mudawarima

**Affiliations:** 1 Department of Rehabilitation, College of Health Sciences, University of Zimbabwe, P.O Box A178, Avondale, Harare, Zimbabwe; 2 Trauma Center Physiotherapy Private Practice, 1 Borrowdale lane, Borrowdale, Harare, Zimbabwe; 3 Department of Rehabilitation, Harare Central Hospital, P.O Box ST 14, Southerton, Harare, Zimbabwe

**Keywords:** Physiotherapy, Cross-sectional study, Patient perspectives, Burn rehabilitation

## Abstract

**Background:**

Physiotherapy is an integral part of treatment for paediatric burns. In Zimbabwe, children are admitted in paediatric burn unit with their caregivers, who play important roles such as providing explanation and obtaining cooperation of the child during physiotherapy, which is often uncomfortable or painful to the patient. The aim of this study was to determine the perspectives of caregivers towards physiotherapy interventions administered to hospitalized children at central hospitals in Harare, Zimbabwe.

**Methods:**

A descriptive cross-sectional study was conducted using self-administered questionnaires. The study was carried out at two large central hospitals (Parirenyatwa Hospital and Harare Central Hospital). The study targeted all the caregivers of children below the age of 12 years with a diagnosis of burns, irrespective of severity or area affected, who were admitted in the two paediatric burn units. Of the 34 caregivers eligible to participate, 31 (91.1 %) questionnaires had complete data and were analysed. The analyses were done using Statistica version 12.0.

**Results:**

The median age of the caregivers was 28 years (IQR = 24–33 years). Female caregivers constituted 90.3 % of the sample. The majority of the caregivers (*n* = 26, 83.9 %) were biological mothers to the hospitalised child. The majority of children (*n* = 20, 64.5 %) hospitalised were between 0 and 4 years. The commonest cause of burns was scalding (*n* = 19, 61.2 %). The burns were mainly in the upper extremities (*n* = 11, 35.5 %). Physiotherapy for the burns was mainly active and passive joint range of motion exercises (*n* = 30, 96.8 %). The caregivers’ perceptions towards physiotherapy were mainly positive (*n* = 20, 64.5 %) indicating that physiotherapy plays an important role in burn management. Of the 21 caregivers given a ward exercise programme, 13 (61.9 %) were not compliant.

**Conclusions:**

Caregivers’ perspectives towards physiotherapy were largely positive and are similar to those found in other studies. The majority of the caregivers indicated that physiotherapy was important in the overall management of burns leading to proper healing of the wounds without complications.

## Background

A burn is generally considered as an injury to the skin or other organic tissue primarily caused by heat, radiation, radioactivity, electricity, friction, smoke or contact with chemicals [1]. Worldwidely, more than 310,000 human beings die each year from burns [2]. This is despite significant improvements in life-support measures, antimicrobial therapy and surgical techniques [3]. Paediatrics are hardest hit, accounting for one quarter to half of all patients hospitalised with burns [4, 5]. Global statistics reveal that the highest rate of paediatric burn admissions is found on the African continent [6]. In Zimbabwe, in particular, burn injuries rank among the top ten causes of outpatient attendances at referral hospitals in all age groups [7]. A longitudinal study on the epidemiology of burns conducted by Mzezewa et al. [8] for over 6 years at two burn units in Harare, Zimbabwe, showed that burns predominantly affect children.

Death is not the only problem that burn victims face as more than a million survivors develop complications which include pain, infections, contractures, hypertrophic scarring and deformities [9]. These problems may have a major impact on their developmental, functional, psychological and aesthetic status into adulthood [10–14]. Because of this, scientific research in paediatric burns over the years has shifted focus from acute care and reducing mortality to health-related quality of life and functional recovery post - burn [10]. In this regard, physiotherapy has traditionally been one of the interventions used in burn management. Working in a multidisciplinary team, physiotherapists focus on patient and caregiver education, scar prevention, hypertrophic scar suppression, management of heterotopic ossification, leukoderma and pruritis, as well as restoration of the patient’s functional capacity, such as full range of motion, muscle strength, and independent mobility and activities of daily living [10, 15–17]. However, successful rehabilitation of paediatric burn patients depends largely on the active participation of the child and the caregiver in the prescribed exercise programmes [10, 14].

In central hospitals in Harare, paediatric burn patients are admitted along with their caregivers, who play important roles in providing explanations, persuading and motivating the burnt children, obtaining their cooperation during physiotherapy which is often uncomfortable or painful to the patient. They also play a crucial role in executing ward exercise programmes outlined by the physiotherapists in charge of the patient. Ward exercise programmes are an essential part of continued care for the burn patient executed in the absence of the physiotherapist in order to maintain therapeutic gains achieved during normal physiotherapy [10, 18]. Caregivers’ compliance with the ward exercise programmes may facilitate a better prognosis for the patient. Given the important role caregivers play in the management of paediatric patients with burns in central hospitals, we set out primarily to explore the perspectives of caregivers towards the physiotherapy care for children admitted with burns in two central hospitals in Harare, Zimbabwe. In order to fully understand this, we had to secondarily investigate the characteristics of burn injuries in children hospitalised in the burn unit in terms of mechanism of injury, diagnosis, cause and location of the burn, time of injury, and physiotherapy treatment administered. The paucity of studies on perceptions towards physiotherapy care of caregivers in Zimbabwe is a significant shortcoming. Caregivers are extended members of the burn treatment team, and their views towards care of their child, especially during physiotherapy, are important for clinicians to understand. With the advent of patient and family-centred approaches to clinical care, details about satisfaction with physiotherapy by caregivers can be used to target areas where improvement is needed [13].

## Methods

### Study design and setting

A descriptive cross-sectional study was conducted. The study was carried out in Harare, Zimbabwe, at Parirenyatwa Group of Hospitals (PGH) and Harare Central Hospital (HCH). The two hospitals are the largest public hospitals in Harare and have the largest paediatric burn units. Physiotherapy forms an integral component of burn management in the hospitals. In these settings, physiotherapists attend to all burn patients from the day of admission to discharge, with the responsibility of minimising the adverse effects caused by the injury in terms of maintaining range of movement, minimising contracture development, maintaining pulmonary hygiene, minimising long-term impact of scarring, and maximising functional abilities. It is a standard protocol in both hospitals that burn patients should be seen by physiotherapists every day and a ward exercise programme be prescribed for the caregivers to execute when the physiotherapist is absent.

### Participants

The study targeted all the caregivers of children below the age of 12 years with a diagnosis of burns, irrespective of severity or area affected, who were admitted in the two paediatric burn units at the time of the study. Contextually, caregivers were regarded as any individual admitted with the child. There were 34 caregivers in the two units at the time of the study. All were invited to participate. Caregivers whose child had received at least two treatment sessions of physiotherapy for the burns were eligible to participate. However, if the child was receiving physiotherapy treatment for another condition co-existing with the burns, those caregivers were excluded. Caregivers were also recruited on the basis that they had witnessed at least two previous physiotherapy treatment sessions.

### Instrument

Self- and interviewer-administered questionnaires were utilised to collect data from the caregivers in the burn unit. The survey questionnaire was specifically designed for this study with questions being derived from literature [19, 20]. The questionnaire was partly premised on the Measure of Processes of Care (MPOC) questionnaire developed in Canada to assess caregivers’ self-reported experiences of family-centred behaviours of rehabilitation service providers [20]. The ad hoc questionnaire had two sections, A and B. Section A: A1 elicited the eight items on socio-demographic details of the caregivers such as age, gender, place of residence, relationship to the admitted child, occupation, marital status and level of education; A2 also elicited eight items on demographic and medical details of the admitted child in terms of age, date of admission, diagnosis, cause and location of the burn, time of injury and type of physiotherapy being administered. Section B presented the list of close-ended questions on perceptions towards physiotherapy and ward exercise programmes. There were 13 questions soliciting for perspectives of the caregivers towards physiotherapy and six on ward exercise programmes. Among other questions, caregivers were specifically asked to indicate whether physiotherapy played an important role in burn treatment or not. Furthermore, open-ended questions were used to elicit reasons for satisfaction with physiotherapy services in hospital.

### Instrument development

Prior to use in the main study, the self-developed English questionnaire was evaluated for content validity by four physiotherapists working in the burn units at the two hospitals using a criteria provided by Davis [20]. The mean years of experience in the burn units for the content experts were 6 years with a standard deviation of 2.4. Content validation was done to ensure that the questionnaire adequately covered all the concepts. The experts were asked to rate each question in the questionnaire for relevance based on a four-point scale (1 = *not relevant*, 2 = *somewhat relevant*, 3 = *quite relevant*, 4 = *highly relevant*) [21]. Then, for each question, the item content validity index (I-CVI) was computed as the number of experts giving a rating of either 3 or 4, divided by the number of experts. This was done to calculate the proportion in agreement about relevance on each question. On the analysis of the results, each question with an I-CVI of >0.80 was accepted as content valid and those with below were either deleted or refined based on recommendations proposed by the experts. Thereafter, the validated English questionnaire with 34 items was translated into Shona, a local language spoken in Harare, using forward and backward translation method. The translation strived for idiomatic rather than word for word translation. Feasibility was assessed in a pilot study using ten caregivers for children with paediatric burns at Chitungwiza Central Hospital (CCH). The hospital is located approximately 30 km south of the capital, Harare. Concurrently, reliability of the questionnaire was established by test re-test method with a one-week interval. The kappa coefficient (k) for the close-ended questions was 0.81 to 1, proving the questionnaire to be reliable. Nevertheless, lessons learnt from the pilot study included the need to administer both the Shona and English questionnaire based on the choice of the participant and the need to administer the questionnaires using interviews for the participants who are illiterate.

### Procedure

The study protocol was reviewed for ethics and approved by the Joint Research Ethics Committee for the University of Zimbabwe and Parirenyatwa Group of Hospitals (JREC, Reference number 264/14), Medical Research Council of Zimbabwe (MRCZ, Reference number MRCZ/B772) and Harare Central Hospital Ethics Committee (Reference number HCHEC 080914/51). Clinical directors and the principal matrons in charge of the Paediatric Burn Units from the two respective hospitals gave institutional approval and permission to have access to patients, caregivers and the patient medical notes.

Data collection was conducted between November 2014 and January 2015. For ease of description, it was divided into three stages. In the preparatory stage, the research team met the principal matrons of each respective burn unit to discuss the procedural issues of the project and agree on the date and time convenient for data collection. It was agreed that data was to be collected in the mornings at all the hospitals before the children had their physiotherapy treatment session for the day. In the intermediate stage, the research team visited the burn units to identify eligible participants and obtain written informed consent from the caregivers.

The last stage involved administering the questionnaires to the caregivers. The questionnaires were either self- or interviewer-administered depending on the level of assistance needed by the participants. To minimise bias, the interviews were standardised for all the participants and were conducted by one member of the research team (VJZ). Data collection took place in the empty cubicles in the respective burn unit. An audit of patient medical notes was also conducted to establish the child’s diagnostic details and the type of physiotherapy treatment being administered.

### Data analysis

Data analysis was carried out using Statistica version 12. Data normality was checked using Shapiro-Wilk test for continuous variables such as age. Data were analysed from the questionnaires that were completely filled in by the participants. Self-administered questionnaires with more than five questions not responded to were discarded from the analysis. Descriptive statistics were used to describe baseline characteristics of the participants and their children. Mann-Whitney *U* test was used to assess for a significant difference in the age ranking between male and female caregivers. Length of hospital stay was calculated as number of days in hospital from admission to the day of data collection. The mean length of hospital stay for the admitted children was computed and a student independent *t* test used to compare between the sexes. Pearson product moment correlation (*r*) was used to check for correlation between length of stay in hospital and severity of burn given as total body surface area (TBSA) burnt. One-way analysis of variance (ANOVA) was used to assess if there was a significant difference in the mean length of stay in hospital by diagnosis. Diagnosis was categorised into four groups as first degree burn, second degree burn, third degree burn and fourth degree burn. Burn severity was captured as TBSA burnt and was categorized into three: <10 %, between 10 and 20 % and ≥20 %. Perspectives of caregivers towards physiotherapy treatment are presented as frequencies. Fisher’s exact was used to assess for association between categorical variables at the level of significance set at *P* < 0.05. Qualitative data collected in the questionnaire using open-ended questions were analysed thematically.

## Results

### Sample characteristics of the caregivers

Of the 34 caregivers eligible to participate, 31 (91.1 %) of the questionnaires were completely filled in and were then analysed. Table 1 shows demographic information of participants. The majority of the caregivers were female (*n* = 28, 90.3 %). The median age of the caregivers was 28 years (interquartile range = 24–33 years). There was a significant difference in the age ranking of the caregivers by gender. Male caregivers were significantly older compared to female caregivers as indicated by the Mann-Whitney *U* test (*Z* = −2.5, *P* = 0.01). The majority of caregivers (*n* = 26, 83.9 %) were biological mothers of the admitted child and were residing in Harare (*n* = 25, 80.6 %). Of the 31 caregivers, the majority were married (*n* = 24, 77.4 %) and 26 (83.9 %) were unemployed. Twenty-four caregivers (77.4 %) had reached secondary school level, and only 2 (6.5 %) had reached tertiary education. The majority of the caregivers (*n* = 10, 32.3 %) had three children in total.

**Table 1:** Socio-demographic characteristics of the caregivers (*n* = 31)

Characteristic	Response	Number	Percent
Gender	Male	3	9.7
	Female	28	90.3
Age (years)	<20	2	6.5
	20–29	15	48.4
	30–39	11	35.5
	>40	3	9.7
Place of residence	Harare	25	80.6
	Outside Harare	6	19.4
Marital status	Married living with husband/wife	24	77.4
	Others^a^	7	22.6
Employment status	Employed	5	16.1
	Unemployed	26	83.9
Educational level	Primary	5	16.1
	Secondary	24	77.4
	Tertiary	2	6.5
Relation to the child	Mother	26	83.9
	Father	3	9.7
	Other^b^	2	6.5
Number of children	1	6	19.4
	2	8	25.8
	3	10	32.3
	4	4	12.9
	5	3	9.7

^a^Refers to participants unmarried, divorced or widowed

^b^Refers to anyone else not biological parents of the child

### Sample characteristics of children

There were 17 (54.8 %) boys and 14 (45.2 %) girls in the sample. The majority of the children (*n* = 20, 64.5 %) with burns were between the ages of 0–4 years. Sixteen (51.6 %) of the children had TBSA burnt covering <10 % whereas 13 (41.9 %) had between 10 and 20 %. The commonest cause of burns in the children was scalding (*n* = 19, 61.2 %). The burns were largely covering the arms in the majority (*n* = 11, 35.5 %) of the children, followed by the trunks and legs. Of the 31 children, most of the burns (*n* = 22, 71.0 %) occurred in the daylight hours compared to the evening or night time. At the time of the study, the majority of the children (*n* = 19, 61.3 %) had been in hospital for 7 to 21 days with different burn diagnoses. Mean length of stay was, however, 15.2 ± 3.2 days at the time of the study. There was a strong positive correlation between length of stay in hospital and the % TBSA (*r* = 0.67). ANOVA showed no significant difference in the mean length of stay in hospital depending on diagnosis (*F* (3, 17) = 1.32, *P* = 0.30).

### Caregivers’ perspectives

Of the 31 caregivers, the majority (*n* = 19, 61.3 %) reported that their child was seen four to five times in the last 7 days by a physiotherapist. Patient medical notes showed that physiotherapy for children with burns regardless of diagnosis mainly included active and passive joint range of motion exercises (*n* = 30, 96.8 %), chest physiotherapy (*n* = 28, 90.3 %), muscle strengthening exercises (*n* = 27, 87.1 %), patient education and ward exercise programme (*n* = 22, 71.0 %), out of bed mobilisation (*n* = 21, 67.7 %), soft tissue massage (*n* = 17, 54.8 %) and posture correction (*n* = 15, 48.4 %).

Twenty (64.5 %) of the caregivers perceived physiotherapy to be important in the overall management of burns. Positive perspectives on the importance of physiotherapy were, however, not related to caregivers’ gender, age, place of residence, employment status and the length of stay in hospital, child’s TBSA and number of physiotherapy sessions done in the last week to the child (Table 2). Of the 20 caregivers who perceived physiotherapy to be important, majority (*n* = 19, 95 %) placed physiotherapy in the context of their child and agreed to strongly agreed that physiotherapy was necessary for the proper healing of their child’s burns. In addition, the majority (*n* = 27, 87.1 %) of the caregivers agreed to strongly agreed noticing benefits of physiotherapy to their child since admission into the burn unit. However, all the caregivers (*n* = 31, 100 %) strongly agreed that they would want their child to continue with physiotherapy when given an option to choose.

**Table 2:** Factors associated with perceived knowledge of caregivers on the importance of physiotherapy in burn management (*n* = 31)

Variable	Response	Perceived knowledge of the importance of physiotherapy, *n* (%)		Total (*n* = 31)	Fisher’s exact value
		Yes (*n* = 20)	No (*n* = 11)	*n* (%)	
Gender	Male	2 (10)	1 (9.1)	3 (9.7)	0.72
	Female	18 (90)	10 (90.9)	28 (90.3)	
Age (years)	<30	12 (60)	5 (45.5)	17 (54.8)	0.48
	≥30	8 (40)	6 (54.5)	14 (45.2)	
Place of residence	Harare	18 (90)	7 (63.6)	25 (80.6)	0.98
	Outside Harare	2 (10)	4 (36.4)	6 (19.4)	
Employment status	Employed	3 (15)	2 (18.2)	5 (16.1)	0.60
	Unemployed	17 (85)	9 (81.8)	26 (83.9)	
Length of hospital stay	<15 days	12 (60)	3 (27.3)	15 (48.4)	0.85
	≥15 days	8 (40)	8 (72.7)	16 (51.6)	
Total body surface area of the child burnt	<10 %	9 (45)	7 (63.6)	16 (51.6)	0.27
	≥10 %	11 (55)	4 (36.4)	15 (48.4)	
Physiotherapy sessions per week	≤3 days	6 (30)	4 (36.4)	10 (32.3)	0.51
	>3 days	14 (70)	7 (63.6)	21 (67.7)	

Twenty-nine (93.5 %) of the caregivers were satisfied to very satisfied with the overall physiotherapy services in the ward for their child. The caregivers’ reasons for being satisfied were sought using open-ended questions. Of the 31 caregivers, 15 (48.4 %) caregivers responded to that question. The two main thematic reasons for satisfaction with physiotherapy services include good professionalism exhibited by the physiotherapists and the fact that physiotherapy was contributing to the child’s general improvement. One participant wrote that ‘My child is improving and getting better, she can now do some of the things that she could not do before treatment’. Another participant interviewed said that ‘My child is healing well without disability’.

The majority of the caregivers (*n* = 23, 74.2 %) and (*n* = 28, 90.3 %) agreed to strongly agreed that physiotherapists explain their treatment techniques to them and provide informative reasons justifying their techniques, respectively. Moreover, the majority of the caregivers (*n* = 26, 83.9 %) agreed to strongly agreed that physiotherapists discuss with them the nature of the child’s condition, prognosis and general improvements. Nevertheless, physiotherapy was thought to induce pain resulting in the child crying during treatment by 10 (32.3 %) of the caregivers. Twenty-eight (90.3 %) of the caregivers did not feel that physiotherapy was time wasting, and 30 (96.8 %) did not feel that physiotherapy was money wasting.

### Ward exercise programmes

Of the 31 caregivers, the majority (*n* = 21, 67.7 %) indicated that they were given a ward exercise programme for their child by a physiotherapist. Of these, 17 (80.9 %) of the caregivers knew about the importance of carrying the ward exercise programme in the context of their child. However, of all the caregivers given the ward programme, 13 (61.9 %) were not carrying out the ward exercise programmes at all.

## Discussion

The primary aim of the study was to explore the perspectives of caregivers towards physiotherapy treatment of children admitted with burns in central hospitals in Harare, Zimbabwe. To the authors’ knowledge, there are no studies specifically related to this matter in the literature. This renders comparisons with other studies difficult. Although this could have limited participants from fully expressing their concerns, most questions used to elicit perspectives were close-ended questions. Future studies using mixed methods and larger sample sizes may provide more useful information. Therefore, this study should be seen as a pilot study describing the baseline opinions of caregivers of children admitted with burns with regard to physiotherapy treatment in hospital. Given that the participants were few and were drawn from two hospitals, the results should be interpreted with caution especially with regard to generalisations to other settings within and outside Zimbabwe. This is a limitation of the study, as the perspectives expressed in this study reflect the views of the caregivers who participated in the study. Nevertheless, the results of this study are consistent with results of other population surveys [22] and the response rate for the study was satisfactory eliminating bias from non-participation. The high response rate could be attributed to immediate collection of the questionnaires after being completed by the participants.

The majority of the caregivers in this study were women as compared to men. Biological mothers to the admitted child provided most of the caregiving in hospital. This probably reflects the Zimbabwean socio-cultural context where mothers are usually left to care for the family especially children whilst fathers run around to support the family financially [23]. Additionally, the majority of the caregivers in this study were unemployed, hence best placed to care for the burnt child in hospital. These findings have been observed by other authors [22, 23]. The caregivers sampled for this study were relatively young with the majority between 20 and 40 years and having a total of three children. In fact, the youngest caregiver in the study was 17 years old, possibly reflecting the existence of early marriages in Zimbabwe. A study conducted in India profiling the socio-demographic characteristics of mothers of hospitalised children in surgical wards showed similar findings [23]. In the present study, burn injuries disproportionately affected the 0–4-year age group more than any other age group for children, a finding partly shared by Kadir [24]. This age group represent individuals who are either completely dependent on their parents/caregivers in their daily life or have started experimenting unwittingly with the environment predisposing them to injuries [25]. Consistent with most of the literature on burns, the most common burn injury was scald due to hot water [4, 25]. The majority of children in the present study were burnt in the daylight hours compared with the evening or night. This may be a multifactorial effect: children tend to more active during the day and also activities such as cooking and bathing are common during the day [4].

### Caregivers’ perspectives

Caregivers of children with burns had more positive than negative perspectives towards physiotherapy treatment of children with burns in hospital. The majority of the caregivers indicated that physiotherapy was important in the overall management of burns leading to proper healing of the wounds without complications. These results support findings from literature which describes physiotherapy as an ‘indispensable’ part of the whole burn care process [26]. This realisation of the importance of physiotherapy by caregivers is crucial in light of the painful treatment techniques commonly used by physiotherapists in burn management. With this knowledge, caregivers can assist in providing explanation, persuading and motivating the burnt child, obtaining their active cooperation with physiotherapy.

In the present study, the majority of the children were receiving physiotherapy on most days of the week (4–5 days) which included mainly a combination of active and passive joint range of motion exercises (stretches), muscle strengthening exercises, chest physiotherapy and soft tissue massage. The variability in physiotherapy treatment for the children with burns could be explained by the highly variable nature of the burns in the sample [26] and the differences in the location of the burns. In the present study, the majority of the children had burns covering less than 10 % TBSA and an almost equal number of children had burns covering between 10 and 20 %. Most children were affected in the arms followed by trunks and legs warranting the use of passive and active joint stretches, muscle strengthening exercise and chest physiotherapy to suppress respiratory complications. Interestingly, all the caregivers were willing to see their child continue with physiotherapy given the option to choose. This could be attributed to the fact that some of the caregivers had started noticing the therapeutic benefits of physiotherapy on their child at the time of the study. Satisfaction with physiotherapy was high among caregivers citing good professional behaviour of the physiotherapists and the fact that the child were improving, a finding shared by Saloojee et al. [20]. Most caregivers felt that physiotherapists explained, giving reasons, their choice of treatment techniques to them.

The majority of the caregivers agreed being given ward exercise programmes by the physiotherapist in charge of their child. This is important for continued care of the paediatric burn patient in the absence of the physiotherapist. However, a sizeable number (13 (61.9 %)) of the caregivers given the ward programme were not carrying out the ward exercise programme at all. Although the reasons behind this were not fully explored, the results showed that about 32 % of the caregivers indicated that physiotherapy made their child cry all the times. This shows that physiotherapy is uncomfortable and painful for some children. Pain and crying are very common during physiotherapy [17] due to the invasive nature of the treatment methods. Treatment techniques such as passive range of motion exercises (stretches) which are mandatory maybe painful to the children. It is possible that the caregivers may be reluctant to induce pain in the child and hence avoid doing the exercises. Pain control becomes essential to make the exercises as easy as possible for the caregivers [17]. However, there is need for the caregivers to be educated on the importance of ward exercise programme and how consequential they are to functional prognosis of the patient [18]. There is also a need for proper training and guidance of the caregivers in executing the ward exercise programmes, especially on techniques that are pain inducing such as active and passive stretches. It also becomes very important to teach caregivers simple ways of relieving the child’s pain after the exercises. As an example, games which incorporate therapy goals such as stretching to catch a ball, reaching and bilateral use of hands depending on the site of injury and therapeutic needs should be encouraged [17]. Of all the caregivers who participated in this study, 21 (67.7 %) admitted being given a ward exercise programme for their child by a physiotherapist. These findings reflect that ward exercise programmes are not always prescribed for every caregiver of paediatric burn patient admitted in our hospital settings. It was beyond the scope of this study to investigate the reasons for that, but this calls for further research aimed at exploring the factors determining the prescription of ward exercise programmes to burn patients by physiotherapists.

## Conclusions

Cognisant of the limitations of the study which included the use of a small purposively selected sample, a questionnaire not robustly assessed for reliability, the use of a quantitative study design to elicit caregivers’ perspectives and the presence of the researcher during data collection, caregivers of children with burns had more positive than negative perspectives towards physiotherapy treatment in hospital. The majority of the caregivers indicated that physiotherapy was important in the overall management of burns leading to proper healing of the wounds without complications. In light of a number of other treatment options available for burn patients such as pharmacological interventions, which could play a significant role in pain management and epithelisation of burn wounds [27], the present study findings should be interpreted cautiously. Given our cross-sectional study findings, there is a need for more studies using robust study designs comparing the effectiveness of physiotherapy in burn wound healing with other pharmacological or non-pharmacological interventions currently used in clinical practice. Factors such as effective pain management and rapid referral to a specialised burn centre have been reported to achieve optimal outcomes in burns [27]. The ward exercise programmes prescribed for continued care by physiotherapists were not followed appropriately. There is a need for proper education of caregivers of the importance of ward exercise programme and how consequential they are to the functional prognosis of burn patient. Future studies are needed to explore level of compliance and the challenges in executing ward exercise programmes experienced by caregivers with children admitted with burns.

## Abbreviations

CCH, Chitungwiza Central Hospital; HCH, Harare Central Hospital; I-CVI, item content validity index; JREC, Joint Research Ethics Committee; K, kappa coefficient; MPOC, Measure of Processes of Care; MRCZ, Medical Research Council of Zimbabwe; PGH, Parirenyatwa Group of Hospital; TBSA, total body surface area
